# O01 Antimicrobial resistance and death certification: are we there yet?

**DOI:** 10.1093/jacamr/dlae136.001

**Published:** 2024-08-23

**Authors:** Ioannis Baltas, Timothy M Rawson, Hamish Houston, Louis Grandjean, Gabriele Pollara

**Affiliations:** Infection, Immunity & Inflammation Department, UCL Institute of Child Health, London, UK; Department of Clinical Microbiology, University College London Hospitals NHS Foundation Trust, London, UK; National Institute for Health Research Health Protection Research Unit in Healthcare Associated Infections and Antimicrobial Resistance, Imperial College London, London, UK; Centre for Antimicrobial Optimisation, Imperial College London, London, UK; David Price Evans Infectious Diseases & Global Health Group, The University of Liverpool, Liverpool, UK; Department of Clinical Microbiology, University College London Hospitals NHS Foundation Trust, London, UK; Infection, Immunity & Inflammation Department, UCL Institute of Child Health, London, UK; Department of Clinical Microbiology, University College London Hospitals NHS Foundation Trust, London, UK; Division of Infection & Immunity, University College London, UK

## Abstract

**Background:**

The impact of antimicrobial resistance (AMR) on death at the patient level remains challenging to estimate. We aimed to characterize AMR-attributable deaths in a large UK centre through death certification.

**Methods:**

This was a retrospective study of all patients who died in University College London Hospitals NHS Foundation Trust in 2022 (*N*=758). Clinical records of participants with positive microbiological samples within 28 days of death were reviewed by two independent investigators. AMR-attributable deaths were defined using a newly proposed standardized patient-level definition after consensus agreement between the two investigators. A third investigator acted as a tiebreaker in cases of disagreement.

**Results:**

Overall, infection was the underlying cause of death for 11.7% (89/758) of participants and was implicated in the pathway that led to death in 41.1% (357/758) of cases. The most common infection syndromes leading to death as documented on the medical certificate of confirmation of death (MCCD) were respiratory tract infections, sepsis and COVID-19. In total, 4.2% (32/758) of all deaths were AMR-attributable. AMR-attributable deaths were more commonly recorded in patients admitted under haematology (7.5%, 9/120) and in younger patients (median age 67 versus 72, *P*=0.01). The median time from the index sample collection until death was 4.5 days (IQR 2–10.5 days). The majority of AMR-attributable deaths (56.3%, 18/32) were caused by treatment failure and subsequent treatment delay caused by intrinsic resistance mechanisms, primarily by *Enterococcus faecium* (38.9%, 7/18), Enterobacterales carrying repressed chromosomal AmpCs (27.7%, 5/18) and *Pseudomonas aeruginosa* (22.2%, 4/18). On the contrary, a minority of AMR-attributable deaths (43.7%, 14/32) were caused by acquired resistance mechanisms, primarily derepressed AmpCs (28.6%, 4/14) and ESBLs (21.4%, 3/14). The median time to effective treatment was 32 h 15 min and did not differ significantly between the two groups. Only 62.5% (20/32) of AMR-attributable deaths as judged by study investigators had infection recorded on the MCCD. AMR was not recorded as a cause of death in any of the patients, including the 14 patients who were deemed to have suffered deaths due to acquired resistance by the study investigators.

**Conclusions:**

Infection and AMR were significant causes of death in this cohort, yet there significantly underreported during death certification. In a low incidence setting for AMR, delays in treatment and AMR-attributable mortality due to intrinsic resistance mechanisms (expected phenotypes) were more common compared with acquired resistance mechanisms.

